# Anticipating, recognising and talking about dying: Can we close the gap?

**DOI:** 10.1016/j.fhj.2026.100510

**Published:** 2026-03-27

**Authors:** Kathryn Mannix

**Affiliations:** Royal College of Physicians of London, UK

**Keywords:** End of life care, Ordinary dying, Communication, Medical education, Public education, Delayed referral, Hidden curriculum

## Abstract

There is a culture of avoidance of discussion of dying in hospital-based NHS healthcare. The disinclination to name, recognise or talk about the possibility of dying among senior staff, and lack of modelling of clear yet compassionate conversations about dying with dying people, communicates a ‘hidden curriculum’ of the low value of this essential component of practice. It results in late recognition of dying, lost opportunities for dying people to reorder their priorities, late referrals for palliative care support, and missed chances to improve quality of life at an earlier stage. It is time to address medical reluctance to view the last stage of life as a precious, unique time that deserves scrupulous attention to what matters most to the sick person; to demedicalise dying; and to work to restore public (and professional) understanding of ‘ordinary dying’.

It is 1982. I am a pre-registration house officer on a combined general medicine and haematology ward for men. It is a Nightingale-style open ward; two long rows of beds face each other, their occupants in progressively greater states of medical instability the closer their bed to the ward entrance, while the most immunocompromised by their condition or its treatment, or those closest to death, are in single rooms adjoining the nurses’ desk: a hierarchy of unwellness.

It is ward-round morning. Our two consultants go round together and use every round as a teaching opportunity. We stop at almost every bed, but we steer quietly past those beds where a family is keeping vigil around a patient deemed to be approaching death. ‘Don’t intrude’, says one consultant, kindly, to the doctors. ‘Comfortable enough?’ he asks the senior nurse on the round, who confirms that medical attention is not necessary. This regular ritual is how I learn that *dying is not doctors’ business*. This is the hidden curriculum.^1^

**Image 1:**
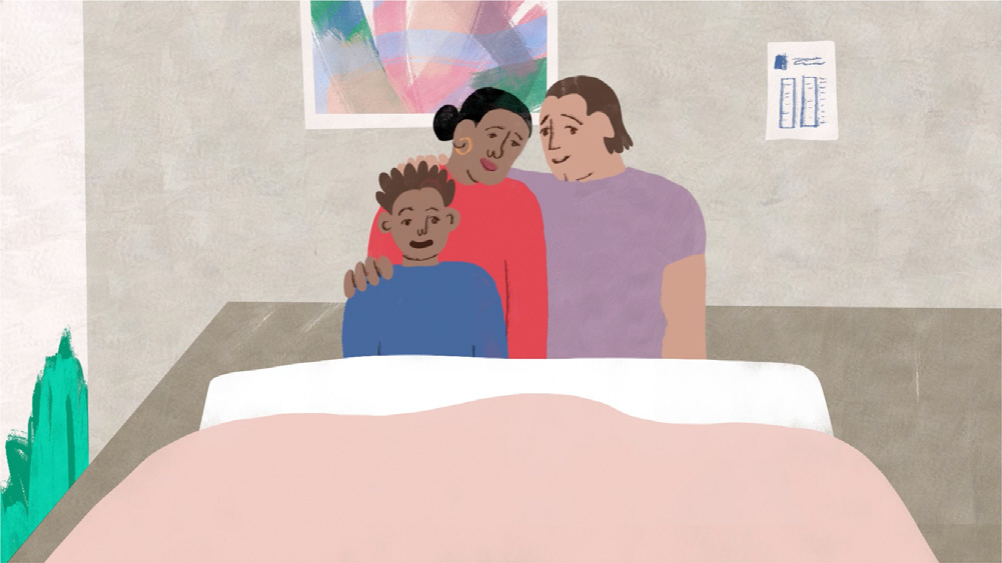
Patient’s view of family at the bedside. Courtesy of Theos/Emily Downe ‘Dying for Beginners’ (reproduced with permission).

Four years, several postgraduate medical exams and many (many) patient deaths later, I am a new trainee in a hospice. A nurse tells my consultant that an elderly patient is afraid of being overwhelmed by pain as she dies. The last 4 years have taught me that *this is not a doctor’s business*. But the consultant takes immediate action, and he requests my company while we address her fears. What on earth can he say? How can we predict what may happen? I am about to be given a masterclass in the art of talking about dying.

In a 15-min consultation, I observed a senior doctor elicit his patient’s concerns about dying, her previous experience of watching someone die, and then describe the process of ‘ordinary dying’ to her (see Box 4): a process she had observed without having received explanations as her husband died in hospital from heart failure a decade previously. The de-catastrophising of her expectations, and the immense comfort she experienced from understanding what lay ahead, was palpable. This never-before-discussed information was the intervention she needed to face her own dying. I have described that perspective-shifting conversation elsewhere.^2^, ^3^

During their conversation, to my astonishment, I recognised everything that my trainer was describing to our patient. I came to understand, for the first time, that dying is a bodily, physiological process that I had witnessed many times without having previously been aware enough to appreciate its phases and stages (see Box 1). I also learned that to speak about dying with candid compassion, at a pace that responds to a dying person’s appetite for information, reduces their fear, enhances their confidence, and displaces their loneliness.

BOX 1 **Signs and symptoms of the dying process.**

Observing the process of dying is as much a matter of pattern recognition as the rest of medicine. This box is based on the author’s experience and on a helpful systematic narrative review of clinician observations.^4^

**Early (weeks–days before death):**

Increasing fatigue and weariness; need for sleep; gradual increase in diurnal sleeping and reduced time awake. May be associated with muddledness on wakening or persisting delirium.

Loss of appetite.

Declining functional status, sometimes rapid.

Symptom relief and other essential medications may now need to be administered by a non-oral route to preserve comfort or prevent withdrawal symptoms.

**Last week of life:**

Reducing consciousness with associated breathing changes: cycles of fast breathing that decelerates to slow with increasing apnoeic pauses; deep breathing with sighing expiration becoming gradually shallower; cycles repeat.

Reducing blood pressure and peripheral perfusion: cold extremities, peripheral cyanosis, skin fragility and mottling.

Purposeless movement; myoclonic jerks (may suggest opioid accumulation).

**Last 24–72 h:**

Muscle relaxation: flaccid limbs, flattening of face, drooping nasolabial folds, inability to close eyes, mouth rests open and tongue may protrude.

Cheyne–Stokes respiration pattern, death rattle, grunting exhalations.

Circulation reduction: impalpable peripheral pulses, reduced urinary output.

Posture changes: extended neck, mandibular movement on inspiration.

**Throughout:**

‘Invisible visitors’: calming (occasionally disturbing) experience of seeing familiar and dear people, often already dead, a phenomenon well recognised by nurses yet rarely discussed with/by doctors.

Lucid moments lasting minutes to hours, though rare, are reported.

Alt-text: Unlabelled box dummy alt text

Only gradually did I come to appreciate that most doctors were then, and still are, as oblivious to the process of ordinary dying as I had been: they do not recognise it and so they do not name it or respond to its discernible progression. They recognise and name only individual components that may vary from dying person to dying person: weariness, loss of appetite, intermittent or progressive muddledness, electrolyte imbalance, blood pressure changes, disinterest in the world beyond immediate loved ones, susceptibility to skin damage, reduced wound healing, dry mouth, clammy skin, failure of individual organs: a catalogue of problems that they address as I had, individually, without seeing the overarching and progressive pattern. Even when patients have reached a stage of illness at which the focus of care is palliative, doctors’ prognostication of survival is inaccurate and inconsistent,^5^ with both over- and underestimates of remaining life expectancy.

After many years of working in a hospital palliative care liaison team, meeting patients for palliative care consultations and listening to their reflections on the information (or lack of it) that they had received from their doctors, it became clear to me that having the confidence to talk about dying, should the conversation turn in that direction, enables clinicians to hold tender and helpful conversations about every aspect of their patient’s concern, whether or not dying is actually discussed. These are the practitioners who enable anticipatory care planning for the end of life to take place in a timely way, a gift that allows patients and families to feel prepared and confident. In contrast, lacking the confidence to discuss dying frequently leads practitioners to avoid any conversation that might ‘go there’, resulting in delayed and inadequate discussion of disease progression, planning ahead, or anticipation of dying: this translates into belated attempts to introduce comfort-directed care that could have provided relief of physical and emotional distress many days or weeks earlier; hesitance to request palliative care consultation, advice or support for their patients; late realisation that dying is approaching both for the patient and for those who matter to them; lost opportunities for families to embrace the final weeks or months of life; and rushed preparations once the final phase of dying commences, including last-minute and often clumsy adjustment of ceilings of intervention, perceived by patients and families as ‘giving up’. These same patterns of communication were noted in RCP-conducted workshops with doctors at all stages of training.^6^ The hidden curriculum lives on: ‘Just don’t mention dying’. All healthcare practitioners who care for people with progressive conditions should be skilled in conversations that describe and de-catastrophise dying and help people to plan ahead.

## Timely conversations

Most deaths in high-income countries are not sudden and unexpected, but come as a result of long-term conditions or illnesses that have progressed despite treatment or are untreatable: almost 60% of deaths in England and Wales 2015–2024 were from dementia, ischaemic heart and cerebrovascular disease, cancers, chronic lung diseases and influenza/pneumonia.^7^ Around 70% of deaths in the UK are of people over the age of 75^7^ and increasing age is associated with multimorbidity,^8^ frailty^9^ and increasing likelihood of death. Surely, then, planning ahead for the anticipatable crises that punctuate many long-term conditions, and for a person’s care preferences should they become sick enough or fragile enough that dying becomes likely, should be part of the care of people living with those long-term conditions? Yet that is not happening.

**Image 2:**
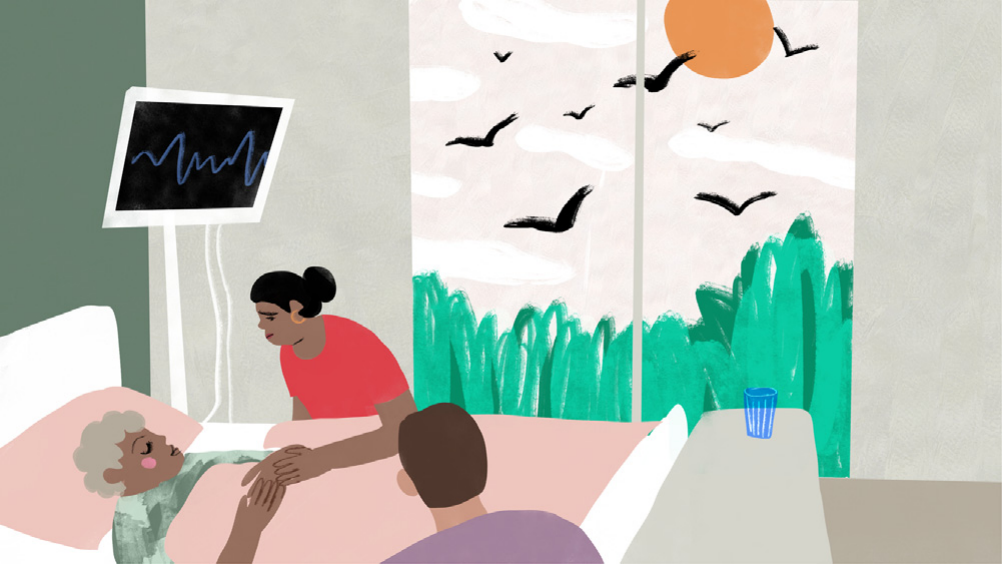
Bedside companions. Courtesy of Theos/Emily Downe ‘Dying for Beginners’ (reproduced with permission).

The 2024 National Audit of Care at the End of Life (NACEL)^10^ reported that, of people admitted to hospital in what turned out to be the admission during which they died, only 18% already had a personal care and support plan. Such plans do not necessarily include end-of-life care planning, but they are intended to allow users to understand their health conditions and likely progression. Palliative care teams repeatedly meet patients with advanced and near-terminal progression of cancers, organ failure or other conditions, referred for palliative care support at a late stage in their illness, who have never previously been offered a discussion about their future care.

Box 2 **Ways into the conversation.**

**1. Flag and invite**

*For a routine review*

‘As well as reviewing your medications/checking your progress at our meeting today, I’d like to use part of our time together to talk about the future, and how you’d like to be looked after when you’re less well than you are now. Would that be alright, or is there someone else you would like to include in a conversation about that?’

**2. Ask a future-focused question**

‘When you think about the future, what do you hope for? And what do you worry about? I find lots of my patients have a ‘best hope’ and a ‘worst dread’ in their mind. If we can discuss yours, that will help us to make sure that whatever happens in future is as close as it can be to your best hope, and as far as we can manage from your worst dread’.

‘Let’s start with your worst dread …’ (this allows us to lift the conversation towards best hope, later on). Listen for ‘… getting worse’, ‘… getting to the end’, ‘… might die’, ‘What if …?’ and ask clarifying questions to be certain that the patient is referring to dying. If unsure, ask

‘How bad do you worry things might get? Do you ever worry that you might die?’ ‘Is that a concern we could talk about?’

Alt-text: Unlabelled box dummy alt text

Without an understanding of the possible impact of their advancing condition on, for example, their mobility or their ease of breathing, a patient cannot make realistic plans for the best place of care. Likewise, without proper discussion it is impossible to offer personalised care respectful of what matters most to an individual whose values may be as diverse as avoiding any drugs with sedative actions to embrace ‘conscious dying’ at the end of life (some individuals, some faiths and cultural groups); being in a place that is personally important or spiritually auspicious; being with loved ones and pets; avoidance of pain; or preference for specific cultural or spiritual rites. Sadly, instead of rounded, careful conversations about their care priorities and the limits of medical intervention, sick people are frequently confronted by an unanticipated and stark discussion of cardiopulmonary resuscitation (CPR) status without any context. The Parliamentary Health Service Ombudsman’s March 2024 report^11^ examines this phenomenon in depth and makes some excellent recommendations, but the difficulty remains that, on admission to hospital, clarification of resuscitation status is required within a short period of time. This task is too often allocated to an inexperienced and busy resident, almost always without senior support, supervision and feedback. The hidden curriculum repeatedly demonstrates the low priority of helping mortally sick individuals to make careful decisions for themselves or to be supported to understand fully the medical decisions that have been made about them. At the same time, the hidden curriculum insists that all deterioration towards the end of life be interpreted as a potential, imminent ‘cardiac arrest’ rather than anticipatable, recognisable and irreversible cessation of breathing and heartbeat, ie dying, that can be accompanied with dignity by offering insightful and meticulous supportive care.

## Revising the hidden curriculum

There will always be a hidden curriculum. Students, trainees, colleagues, team members all notice and interpret the attitudes of their senior colleagues, teachers, trainers and supervisors. What is spoken of, and what is avoided? Their respect, reverence or disdain for specific topics, for patient status, for colleagues in other specialties. This includes the way that dying people are spoken to, the way that death is spoken of, and the way that colleagues who specialise in end-of-life care are valued in the healthcare system.

The equivalent of the kindly tiptoe past the bed still takes place in multidisciplinary team meetings, where time pressures dictate that dying patients take low priority for discussion, and a person who ‘is palliative now’ becomes shorthand for ‘is no longer of interest to us’.^6^ Meanwhile, in a crisis of NHS bed availability, other dying people may still be waiting in corridors to be allocated a ‘home’ team with no continuity of care, a situation that neglects patient needs and further demoralises overstretched staff.

The value of specialist palliative care services, both to offer care and palliative treatments to patients and to offer advice and support to non-specialist colleagues, is demonstrated in the under-commissioning of services that remain stretched and unable to offer 24/7 support around the UK;^12^ services that continue to rely on public support and charitable donations to provide care that underpins the only phase of life that is guaranteed to arrive: dying. What does that tell us all about the way that dying people are valued by society?

Revising the hidden curriculum about dying requires a systemic change in attitude that recognises the vulnerability and right to excellent care of everyone approaching the end of life. This includes investment that recognises and addresses inequities in provision and accessibility of services; co-design with communities to commission services that are of value to them instead of applying a single design template based on 20th century, White, educated middle-class ideas; appreciation that dying is everyone’s business (every community prepared to help)^13^; and restoration of public understanding of dying. All of this, of course, means finding better ways to talk about dying.

## Talking about dying

There are excellent, validated communication skills packages available to clinicians that promote compassionate conversations about the direction of care and levels of intervention; these include the Serious Illness Conversation,^14^ the Dignity Question and Dignity Therapy,^15^ Real Talk conversations training,^16^ and the communication skills guidance included in NHS Scotland’s ‘Realistic Medicine’ programme,^17^ with new programmes under development to supplement and fill gaps identified in extant programmes, eg All providers Better Communication Skills (ABCs).^18^

Box 3 **Continuing the conversation.**

**1. Mention of dying by a patient is the beginning, not the end, of a conversation. Pick it up and be curious about expectations and concerns.**

‘So, you’ve had thoughts about dying / the end of your life / what might happen towards the end? When you think about that, what goes through your mind? Can you tell me more about that? What’s the best you hope for about it? What worries you about it?’

In response to what worries a person, even if it is an unlikely concern, stay with it and remain curious. It is helpful to allow concerns to be expressed and explored fully before moving on to correcting misperceptions or exploring more likely scenarios.

**2. Follow up a cue**

‘It sounds as though what you’re expecting is far worse than anything we’re expecting for you. I hear that a lot. Most people find that knowing a bit more about what to expect puts their mind at ease. When you feel ready, could I tell you a little bit more about what we might expect to happen towards the end of your life, and how we’ll work together to ensure you’re comfortable enough?’

**3. Approach from another perspective**

‘My patients sometimes worry about what their family might see and hear once dying has started. Families and friends find it all less stressful if they have an idea what to expect. I can describe that to you, or to them, or to all of you together. Shall we find a time to do that?’

Alt-text: Unlabelled box dummy alt text

Talking about dying, however, is a two-way process, and reliable, accurate and accessible information for the public is also critical to levelling up the conversation about end-of-life care. A variety of initiatives that seek to encourage public awareness and insight into mortality, living the last part of life well, expressing opinions about future care and taking ownership of the process of dying as an intimate, personal and private time of an individual’s life rather than a medical event, are welcome developments; examples include Dying Matters;^19^ Good Life, Good Death, Good Grief;^20^ and the End Well Project;^21^ and the growing number of end-of-life doulas,^22^^,^^23^^,^^24^ members of the public trained to offer companionship and support to individuals and their families who are facing the end of life. My experience as the author of a public-facing book about dying^2^ is that there is public hunger for good and reliable information, and high levels of engagement rather than the supposed ‘taboo’ so frequently spoken of.

Box 4 **Describing ‘ordinary dying’.**

This 4-min video describes the process of dying. It was commissioned by Theos Think Tank and is reproduced here with their permission. It is used widely by palliative care professionals and others around the world to help dying people and their families to understand the process of dying.

https://www.theosthinktank.co.uk/comment/2023/10/25/dying-for-beginners

**Image 3**. Title image of Theos ‘Dying for Beginners’ Video (Theos/Emily Downe, reproduced with permission).

Alt-text: Unlabelled box dummy alt text

## Conclusion

For as long as the process of dying remains hidden, ignored and not spoken of, it will conjure fear and generate avoidance in a self-perpetuating cycle. Dying is, simply, human destiny. Its inevitability, and its usual physiological processes, should be a topic of public education and discussion, and of thoughtful commissioning of appropriate services that support the care and comfort of citizens living the last part of their lives. Dying is not ‘owned’ by medicine, but our patients should expect all their doctors to be familiar with its course, confident in its discussion, and skilled in its accompaniment.

## CRediT authorship contribution statement

**Kathryn Mannix:** Writing – review & editing, Writing – original draft, Conceptualization.

## Declaration of Competing Interest

The authors declare the following financial interests/personal relationships which may be considered as potential competing interests: Dr Kathryn Mannix is the author of *With the end in mind*, a non-fiction book for general public consumption, that is relevant to and cited in this paper. Dr Mannix receives royalties on sales of this book. If there are other authors, they declare that they have no known competing financial interests or personal relationships that could have appeared to influence the work reported in this paper.
